# Modeling radiofrequency responses of realistic multi-electrode leads containing helical and straight wires

**DOI:** 10.1007/s10334-019-00793-9

**Published:** 2019-11-19

**Authors:** Mikhail Kozlov, Marc Horner, Wolfgang Kainz

**Affiliations:** 1 Max Planck Institute for Human Cognitive and Brain Sciences, Stephanstrasse 1a, 04103 Leipzig, Germany; 2 ANSYS, Inc, Evanston, IL USA; 3 Division of Biomedical Physics, Office of Science and Engineering Laboratories, U.S. FDA, CDRH, Silver Spring, MD USA

**Keywords:** Computational modeling, RF simulations, Tissue heating, Implanted medical device, Finite element method (FEM)

## Abstract

**Purpose:**

To present a modeling workflow for the evaluation of a lead electromagnetic model (LEM) consisting of a transfer function (TF) and a calibration factor. The LEM represents an analytical relationship between the RF response of a lead and the incident electromagnetic field. The study also highlights the importance of including key geometric details of the lead and the electrode when modeling multi-electrode leads.

**Methods:**

The electrical and thermal responses of multi-electrode leads with helical and straight wires were investigated using 3D electromagnetic (EM) and thermal co-simulations. The net dissipated power (*P*) around each lead electrode and the net temperature increase at the electrodes (Δ*T*) were obtained for a set of incident EM fields with different spatial distributions. A reciprocity approach was used to determine a TF for each electrode based on the results of the computational model. The evaluation of the calibration factors and the TF validation were performed using the linear regression of *P* versus the LEM predictions.

**Results:**

*P* and Δ*T* were investigated for four multi-electrode leads and four single-electrode leads containing either helical or straight wires. All electrodes of the multi-electrode lead were found to be points of high power deposition and temperature rise. The LEMs for the individual electrodes varied substantially. A significant dependence of the calibration factors on the surrounding tissue medium was also found. Finally, the model showed that the TF, the calibration factor, *P* and Δ*T* for multi-electrode leads differ significantly from those for single-electrode leads.

**Conclusion:**

These results highlight the need to evaluate a LEM for each electrode of a multi-electrode lead as well as for each possible surrounding medium. It is also shown that the results derived from simulations based on simplified single-electrode leads can significantly mislead multi-electrode lead analyses.

## Introduction

Radiofrequency (RF)-induced heating of tissues near an electrode of an active implantable medical device (AIMD) is a potential problem for patients undergoing magnetic resonance imaging because tissue damage may occur for sustained exposure above critical temperatures (MRI) [1–5]. Two quantities are typically used to characterize the RF responses of an AIMD: (i) the net dissipated power (*P*) surrounding the AIMD lead electrode and (ii) the net temperature increase at the electrode (Δ*T*):

1 $$P = \mathop \int \limits_{ }^{{{\text{HSIV}}}} \left( {\sigma \cdot \left| {E_{{{\text{total}}}} \left( v \right)} \right|^{2} - \sigma \cdot \left| {E_{{{\text{backgnd}}}} \left( v \right)} \right|^{2} } \right) \cdot {\text{d}}v,$$

2 $$\Delta T = \Delta T_{{{\text{total}}}} - \Delta T_{{{\text{backgnd}}}} ,$$

where *σ* is the electrical conductivity of the surrounding medium, *E*_total_(*v*) is the electrical field with the lead in place, *E*_backgnd_(*v*) is the electrical field without the lead in place, HSIV is the hot spot integration volume, Δ*T*_total_ is the temperature increase at the electrode with the lead in place, and Δ*T*_backgnd_ is the temperature increase at the electrode without the lead in place.

In vivo measurement of *P* and Δ*T* during an MRI scan of live human subjects is not currently feasible. Δ*T* has been evaluated in a limited number of cadaver [6] and animal [7, 8] studies, however. Computational modeling has been identified as a useful approach for understanding the nature of lead interactions with incident electromagnetic (EM) fields in vivo [9]. Recent publications have analyzed leads containing only one electrode [10–12], in spite of the fact that multi-electrode leads are more common in AIMDs. Multi-electrode leads have also been analyzed [13–18], however substantial simplifications of the lead wire structure were made, e.g., substitution of a multi-wire design with a single wire. These geometric simplifications are a concern because they can have a significant impact on safety assessments.

In spite of continually expanding computational capacity, a prohibitive amount of time is required to conduct electromagnetic (EM) simulations that comprise the full parameter matrix formed by (i) a set of high-resolution human body models that represent the AIMD patient population, (ii) the detailed AIMD model at all possible locations, (iii) a set of MRI RF transmit coils that represent clinically relevant cases, and (iv) a set of relevant patient landmark positions inside the MRI RF transmit coils. Domain decomposition is a common technique to split the solution of a complex problem into a set of substantially simpler sub-tasks. For AIMD RF safety assessments, one possible decomposition consists of separating the human RF exposure due to the MRI RF transmit coil from the assessment of the RF responses of the lead. The former could be accomplished by generating a set of clinically relevant incident tangential RF electric fields (*E*_tan_(*l*)) along the lead pathways, and the latter by evaluating *P* and Δ*T* to this set of *E*_tan_(*l*).

Experimental or numerical domain evaluation of lead RF responses to the full set of *E*_tan_(*l*) derived from human RF exposure analysis is still an extremely complex task because (i) the evaluation should represent the matrix of multi-tissue environments that may be encountered by the lead tip in vivo and (ii) the generation of *E*_tan_(*l*) with complex spatial distributions of amplitude and phase requires the construction of a multi-channel RF transmit source with difficult to implement near field directivity of antennas and low mutual coupling between antennas.

The analytical lead electromagnetic model (LEM) was proposed to evaluate the RF responses to a set of clinically relevant *E*_tan_(*l*) in humans [19]:

3 $$P = A \cdot \left| {\mathop \int \limits_{0}^{L} S\left( l \right) \cdot E_{{\tan}} \left( l \right) \cdot {\text{d}}l} \right|^{2} ,$$

4 $$\Delta T = A_{{\text{T}}} \cdot \left| {\mathop \int \limits_{0}^{L} S\left( l \right) \cdot E_{{\tan}} \left( l \right) \cdot {\text{d}}l} \right|^{2} ,$$

where *A* and *A*_*T*_ are the calibration factors, *S*(*l*) is the transfer function (TF), and *L* is the lead length. Note that the TF is the same for all RF responses of a given lead electrode because the TF is a relative measure of different lead segment contributions in the EM field radiated by a given electrode [20]. Thus, for a given AIMD electrode, the LEM of an RF response consists of the same TF and a calibration factor specific to this RF response. While concepts for developing a LEM have been published, there have been no publications outlining procedures for assessing a LEM of clinically relevant AIMDs. Indeed, the details are considered trade secrets and are highly guarded by AIMD manufacturers.

Several approaches to determine the TF have been published: piecewise excitation [19], reciprocity [21], transmission line modeling [22, 23], and MRI-based [24]. Evaluation of the calibration factor was rarely described, for example [20, 25]. However, it is impossible to evaluate an RF response of a lead for clinically relevant *E*_tan_(*l*) without knowledge of the calibration factor. Both experimental and numerical TF determination procedures have deficiencies. For example (i) it is impossible to define a piecewise excitation with infinitely short length and (ii) some assumptions of the transmission line model are not valid for all lead types. Thus, the measured or simulated TF should be validated using an approach that is independent from the TF generation procedure (a typical requirement of model validation).

The TF can be obtained for multi-tissue lead environments. However, different lead pathways result in different multi-tissue lead environments and thus varied TFs. To avoid multi-tissue testing requirements, the ISO/TS 10974:2018 Tier3 procedure [26] suggests testing in a homogeneous medium with the electrical properties close to those of the medium (tissue) that is in predominant contact with the AIMD. ISO/TS 10974:2018 also specifies that the AIMD should also be evaluated in several media with appropriate electrical properties if more than 10% of the cumulative physical length of the AIMD pathway spans different tissues.

Following from the above and the facts that (i) the measurement results are rather noisy due to substantial uncertainty of measurement probes and other uncertainties of the measurement setups, and (ii) the high cost of a typical measurement setup (greater than $500,000 US), the main goals of this study were to develop a numerical workflow for modeling the LEM and the RF responses of various multi-electrode lead designs using EM and thermal co-simulation of the entire lead and electrodes. The LEM and RF responses for a range of incident EM fields were evaluated in several tissue media. The calculated RF responses, i.e., *P* and Δ*T*, were used to validate the TF and to estimate the correspondent calibration factors. The impact of simplifying a multi-electrode lead to a single electrode was also analyzed.

## Methods

### Computational model overview

The computational modeling workflow consists of four major steps: (i) obtaining the TF for a given lead, (ii) validating the TF and evaluating the calibration factors of the LEM, (iii) evaluating the power deposition and temperature responses to a set of incident EM fields, and (iv) estimating the power deposition and temperature response to the power injection into a multi-electrode lead. 3D EM simulations at 128 MHz were performed using the frequency-domain solver of ANSYS HFSS (ANSYS, Inc., Canonsburg, PA, USA). Volume and surface losses from the 3D EM simulations were input as thermal sources for the thermal simulations performed using the ANSYS Non-Linear Thermal (NLT) platform (ANSYS, Inc., Canonsburg, PA, USA)*.*

The computational meshes of the 3D EM and thermal numerical domains were independently generated in each solver to ensure the best suitable mesh for each simulation modality. A mesh adaption procedure in HFSS increased the number of mesh elements until the variation of *P* or ‖*S*‖_max_ (representing the maximum of the transfer function magnitude ‖*S*‖ over all spatial points) between two consecutive meshes was less than 3%. Applying this procedure resulted in a mesh size in the region of maximum power deposition that was less than 5 μm. The initial temperature for both the implant and the surrounding medium was 22 °C. The initial time step of the thermal simulation was 125 µs, which is more than 10 times shorter than the shortest thermal constant in the simulation domain. The flux convergence of the thermal solver was set to 0.0001. The convergence of the thermal simulation was obtained by manual refinement of the mesh until the difference between maximum temperature rise (Δ*T*_max_) was less than 3% for two sequential meshes. This resulted in a mesh size in the region of maximum thermal gradient that was less than 8 μm.

### Lead description

The exact dimensions and other geometric details of commercially available multi-electrode leads have not been published. We therefore construct the leads used in these studies from literature information [27] as well as publicly available pictures and datasheets. The wire design was estimated based on literature data: (i) the wire diameter is typically less than 0.2 mm and (ii) the distance between adjacent helical wires typically ranges from one to three wire diameters. For mechanical reliability reasons, commercial leads are fabricated using a multi-filament wire. Computational models of multi-filament wires require significant computational resources because each filament must be meshed independently to capture its micrometer-range geometric structure. In our study, the multi-filament wires were substituted with single-filament wires of equivalent cross-section and geometry, namely helical or straight. Mo et al. [28] reported similar temperature rise measurements for leads with seven-filament and bare wires, which indirectly confirms this simplification.

**Fig. 1:**
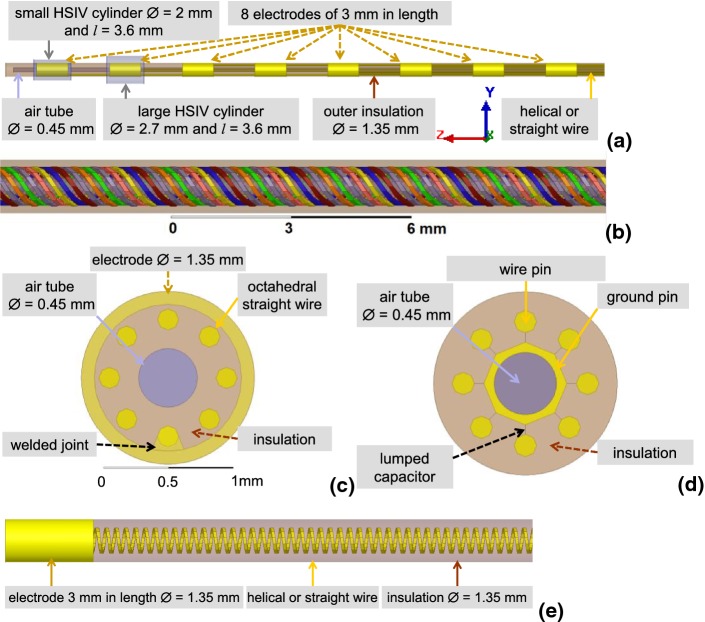
**a** Close-up view of the 8-electrode portion of the lead and hot spot integration volumes (HSIV). **b** Close-up view of the 8 helical wires. **c** Cross-section of the straight-wire lead at the solder location of the wire and electrode. **d** Cross-section of the straight-wire lead in the connector pins region (i.e., proximal end). **e** Close-up view of the electrode portion of the single-electrode lead

Four 8-electrode lead designs were investigated. The leads were 300 mm in length and 1.35 mm in diameter. Each of the eight titanium alloy electrodes was 3 mm in length with outer and inner diameters of 1.35 mm and 1.15 mm, respectively. The distance between the electrodes was 4 mm (Fig. 1a). The solder connection between the wires and electrodes was modeled as a welded joint that was 0.75 mm in length and 0.2 mm in width at the distal end (Fig. 1c). And while not present in commercial leads, an artificial connector of 1 mm in length was employed at the proximal end of the lead to define realistic impedance between wires (Fig. 1d). This connector consisted of 8 wire pins that were in electrical contact with corresponding lead wires, ground pin, and 8 lumped 3.6 pF capacitors installed between the ground pin and wire pins. These capacitors modeled the input impedance of the implanted pulse generator. An internal air tube with diameter of 0.45 mm was located inside all four leads (Fig. 1c). For leads constructed with straight titanium alloy wire, the thickness of each wire was 0.165 mm (Fig. 1c). For leads constructed with helical titanium alloy wire, each helical structure was made of 0.1 × 0.1 mm rectangular wire with a pitch of 2.0 mm and an external diameter 1 mm (Fig. 1b). The distance between adjacent helical wires was ~ 0.15 mm. The relative electric permittivity of the insulator material (*ε*_r_) was varied from 2.7 to 5.5 to investigate the effect of this design parameter on lead performance.

The leads were surrounded by one of three tissue media in each simulation: (i) *ε*_r_ = 78 and *σ*  = 1.2 S/m (approximating blood); (ii) *ε*_r_ = 78 and *σ* = 0.47 S/m (approximating generic biological tissue, as defined in ASTM 2182-11a and ISO/TS 10974:2018 to represent the global weighted average of human tissues), and (iii) *ε*_r_ = 44 and *σ *= 0.354 S/m (approximating nerve tissue) [29]. Values of the electrical and thermal properties for all materials are summarized in Table 1.

**Table 1:** Material properties used in the simulations

Material	Relative electrical permittivity *ε*_r_, [1]	Electrical conductivity, S/m	Specific heat *c*, J/(kg·K)	Isotropic thermal conductivity *k*, W/(m·K)	Density *ρ*, kg/m^3^
Blood [29]	78	1.2	4181	0.6	1001
Average biological tissues	78	0.47	4181	0.6	1001
Nerve tissue [29]	44	0.354	3613	0.49	1075
Titanium alloy	1	0.595 M	526.3	6.7	4430
Low *ε*_*r*_ insulation material	2.7	24 μ	1000	0.2	1350
High *ε*_*r*_ insulation material	5.5	24 μ	1000	0.2	1350

Single-electrode leads were also investigated and compared to the results for the multi-electrode leads. Many dimensions of the single-electrode lead matched those of the multi-electrode lead, including the length and diameter of the lead as well as the length and inner/outer electrode diameters (Fig. 1e). The helical wire design was constructed from eight 0.1 × 0.1 mm rectangular wires with a pitch of 0.33 mm, resulting in a similar distance between adjacent wire turns in the single and multi-electrode leads. Eight different lead designs were evaluated, four with helical wires and four with straight wires. The four helical wire designs were formed from all four combinations of external wire diameter (0.9 mm and 1.1 mm) and insulator relative permittivity (2.7 and 5.5). Similarly, the four straight wire designs were formed from all four combinations of wire diameter (0.73 and 1.0 mm) and insulator permittivity (2.7 and 5.5). The single-electrode leads were capped at the proximal end. The single-electrode leads were only analyzed for the medium with electrical properties approximating blood, i.e., *ε*_r_ = 78 and *σ* = 1.2 S/m because the maximum *P* for multi-electrode leads was observed in this medium.

### Transfer function development and calibration factor evaluation

**Fig. 2:**
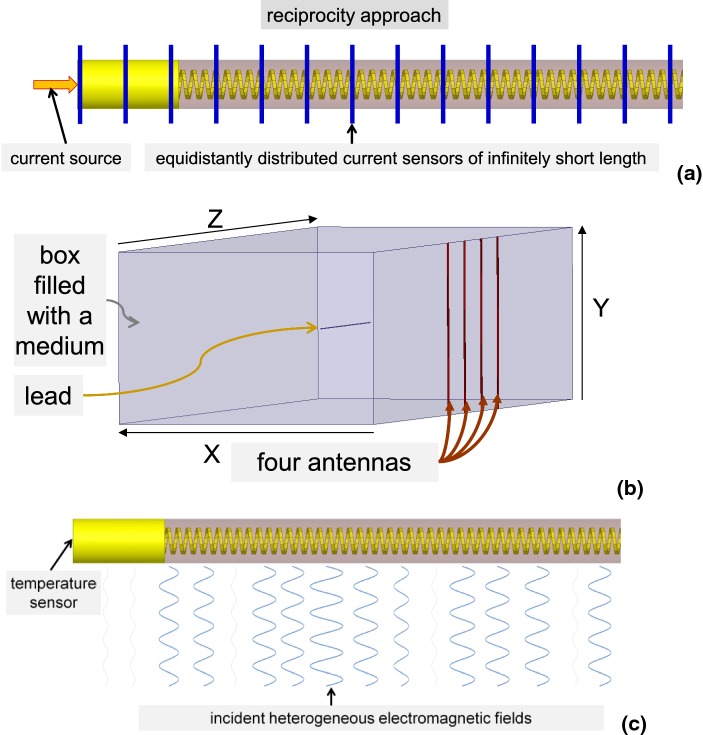
**a** Sketch representation of the TF evaluation. **b** 3D EM numerical domain for evaluation of the calibration factors and LEM validation. The box (600 mm × 400 mm × 2400 mm) is filled with tissue-simulated medium. **c** Sketch representation of lead EM exposure inside the box

Applying the reciprocity approach and using a current source and 161 numerical current sensors of infinitely short length, the TF was obtained at 161 equidistant points (Fig. 2a). The TF was obtained for each electrode, lead, and surrounding medium using this approach. Each TF was normalized such that $\mathop \smallint \nolimits_{0}^{L} \left| {S\left( l \right)} \right| \cdot {\text{d}}l$ = 1 and the phase at the first location was shifted such that the phase of *S*(0) = 0.

Ideally, calibration factor evaluation and validation of the TF at *n* equidistant points requires a set of *n* orthogonal non-homogeneous *E*_tan_(*l*) and corresponding RF response measurements, i.e., *P* or Δ*T*. In reality, calibration factor evaluation and TF validation is accomplished using a linear regression analysis based on the results obtained for a set of artificial heterogeneous *E*_tan_(*l*). This set of heterogeneous *E*_tan_(*l*) is typically generated in a phantom filled with tissue simulating medium by (i) modifying the lead path within the phantom located inside a RF birdcage coil [26] or (ii) modifying the excitation of a dual-channel RF birdcage coil containing the phantom [30]. These approaches require a curved lead trajectory, which can lead to EM field scattering for certain AIMD lead designs and distances between AIMD lead segments. The scattered EM field from one AIMD lead segment to another should be avoided for reliable LEM validation because the scattered EM field modifies the incident EM field. Because (i) to generate computer-aided design (CAD) models of a helical wire for curved lead trajectories is challenging and (ii) a straight lead trajectory results in the smallest EM field cross-scattering, our previously developed numerical approach to obtain a series of heterogeneous *E*_tan_(*l*) for straight lead trajectories [25] was applied as follows.

Each lead was positioned parallel to the z-axis in the middle of a rectangular box (600 mm × 400 mm × 2400 mm) containing one of the three tissue media (Fig. 2b). The box was surrounded by a perfectly matched layer. Four numerical antennas were located along one (*yz*) side of the box, generating an EM field at 128 MHz. Each antenna generated an EM field so that the *z*-component of the electric field was dominant at the lead location. 40 non-uniform *E*_tan_(*l*) were generated using a set of 40 different antenna source amplitudes and phases (Fig. 2c). None of the *E*_tan_(*l*) was a scaled replica of other *E*_tan_(*l*) in the set. *E*_tan_(*l*) along the lead trajectory, $E_{{{\text{backgnd}}}} \left( v \right)$, and Δ*T*_backgnd_ were calculated for the three media without the lead in place.

Two HSIVs were defined: one small and one large (Fig. 1a). Because the evaluation of *P* was significantly faster than for Δ*T*, TF validation was performed using the linear regression of *P* calculated for the small HSIV versus the LEM predictions for the 40 non-uniform *E*_tan_(*l*) as follows:

5 $$\hat{y}_{i} = \left| {\mathop \int \limits_{0}^{L} S\left( l \right) \cdot E_{{{\tan}_{i} }} \left( l \right) \cdot {\text{d}}l} \right|^{2} ,\;i = \{ 1:40\} .$$

Note that the smaller HSIV was selected for TF validation to minimize the influence of the scattered EM field. The larger HSIV was used for evaluation of the correlation between *P* and Δ*T*. For the thermal analyses, Δ*T* was analyzed after 10 and 200 s of continuous excitation. Δ*T* for each electrode was equal to the maximum temperature observed at the electrode external surface. The influence of the medium thermal conductivity on temperature distribution around the electrodes and hot spot determination based on Δ*T* was evaluated by comparing results at 10 and 200 s.

Another output of the linear regression is the linear coefficient of determination (R^2^), which is the quotient of the variances of the fitted values and the observed values of the dependent variable. If R^2^ = 1, all of the data points fall perfectly on the regression line. If R^2^ = 0, the dependent variable accounts for none of the variation in the observed data. In our case, two reasons that may result in R^2^ < < 1 are either that the obtained TF is not correct or the LEM is not the right approach for evaluation of the given RF response with a low uncertainty. In our study, the acceptance criterion for TF validation was R^2^ > 0.95.

The influence of the lead design and the surrounding medium was analyzed using the numerically validated LEMs. It is impossible to specify clinically relevant AIMD trajectories inside the human body and to obtain a set of clinically relevant *E*_tan_(*l*) without indicating a particular type of AIMD. However, the artificial set of 40 *E*_tan_(*l*) distinct from the original 40 *E*_tan_(*l*) supports an initial comparison of *P* and Δ*T* for leads with different designs and surrounding media.

The comparison of results for multi-electrode and single-electrode leads was performed based on Δ*T* at *t* = 200 s. The same set of *E*_tan_(*l*) was used in the investigation of all leads. Maximum values of Δ*T* observed in the 3D EM and thermal co-simulation results were compared.

### Power injection into leads

There is no standard that defines a safe thermal exposure for each human tissue. Animal studies are one approach to confirm a no-harm condition for a given level of RF-induced heating. However, it is challenging to conduct animal studies in RF exposure environments, e.g., a birdcage of an MRI scanner or an RF exposure system. The major difficulty with performing safety evaluations in animals is to generate clinically relevant distributions of amplitude and phases of *E*_tan_. This is because of: (i) substantial differences in the incident EM field generated in humans versus study animals; (ii) difficulty implementing lead trajectories in animals that result in the largest Δ*T* in humans. Experiments in an MRI environment also require the use of expensive non-magnetic tools and MRI- and RF-compatible temperature probes.

A power injection approach was proposed to address the aforementioned challenges in animal studies [26]. This approach should ensure that the power injection from a power source at the proximal end of the lead results in temperature rises encountered during RF exposure and at relevant distributions of amplitude and phases of *E*_tan_. Large inductance-related losses occur in helical wires if the power injection is performed using RF frequencies typical for commercial 3 T MRI scanners (123–128 MHz). Power injection at 0.5 MHz eliminates the lead inductance-related losses and can generate the RF-induced heating in close proximity to an electrode similar to the radiated case at MRI frequencies.

We modeled power injections by connecting a 50 Ω RF power source across the wire pins and the ground pin. The 8 lumped capacitors were removed from the lead model. Continuous injection with *P*_in_ = 0.1 W was applied at 128 MHz and at 0.5 MHz. The electrical properties of the medium approximating blood and nerve tissue were adjusted to those for 0.5 MHz using the IT’IS material property database [29]. Variation of electrical properties for the generic tissue medium is not included in the database and was not applied.

## Results

### Transfer function and calibration factor results

**Fig. 3:**
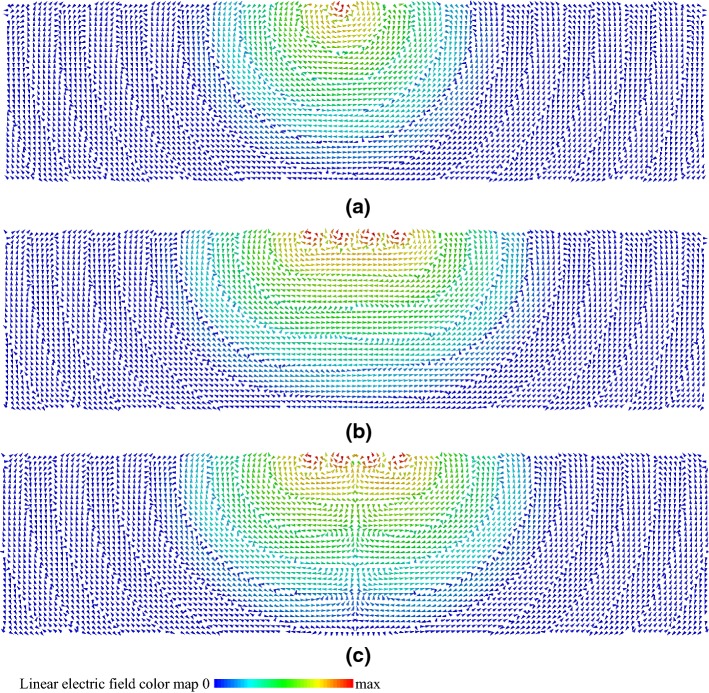
Vector plot of the electric field in the *xz* plane. **a** Excitation of one antenna. **b** Excitation of all antennas simultaneously with the same source amplitudes and phases. **c** Excitation of all antennas simultaneously with the same source amplitudes, but the phase of the first pair of antennas offset 180° from the second pair of antennas

As shown in Fig. 3, the electric field at the location of the leads, and therefore *E*_tan_(*l*), was affected by modification of antenna positions as well as the amplitudes and phases of the RF sources. The influence of the lead on antenna RF field generation was negligible because the leads were located approximately one wavelength from the antennas in the lossy medium. Also, the box was sufficiently large enough in the *z*-direction to minimize the influence of the perfectly matched layer on field propagation from the antennas to the lead location.

**Fig. 4:**
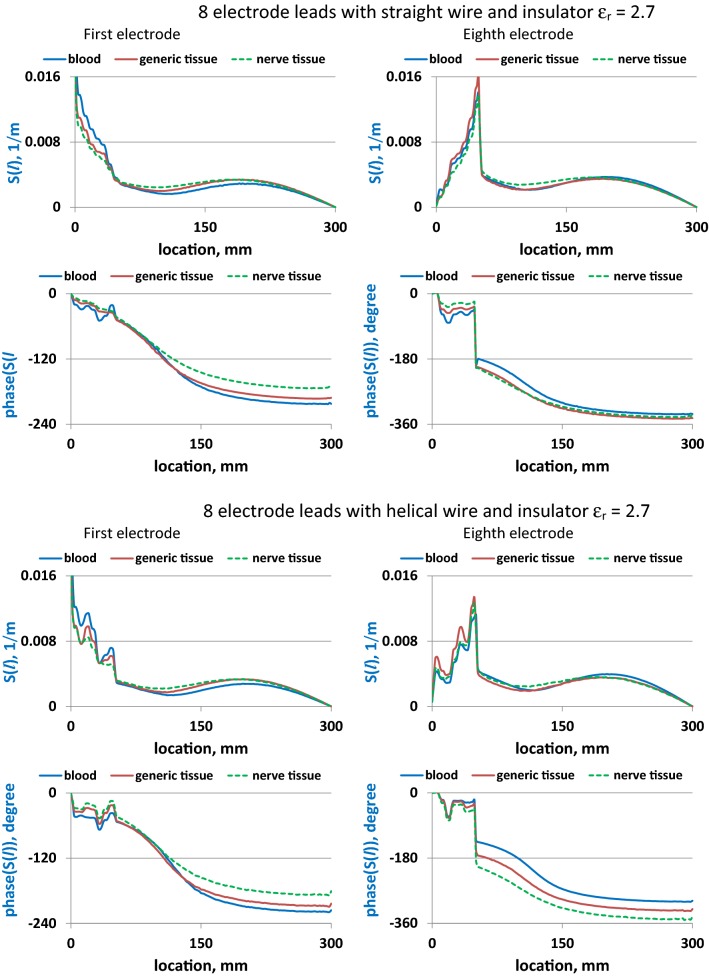
Amplitudes and phases of *S*(*l*) for leads with the straight and helical wires implanted in blood, generic tissue, and nerve tissue

**Fig. 5:**
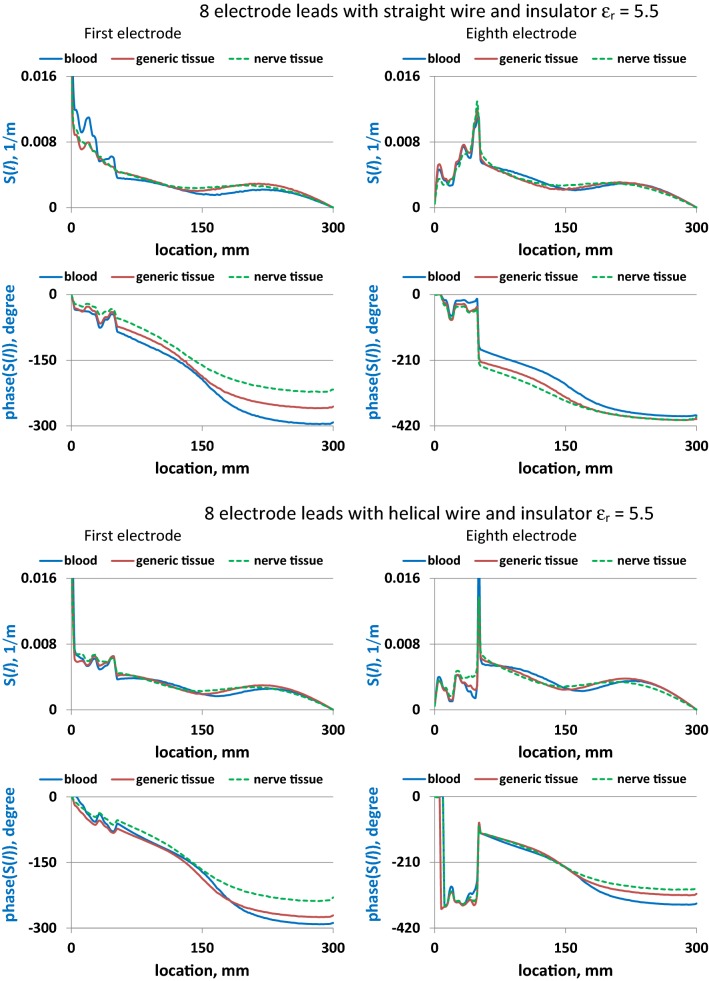
Amplitudes and phases of *S*(*l*) for leads with the straight and helical wires implanted in blood, generic tissue, nerve tissue

**Fig. 6:**
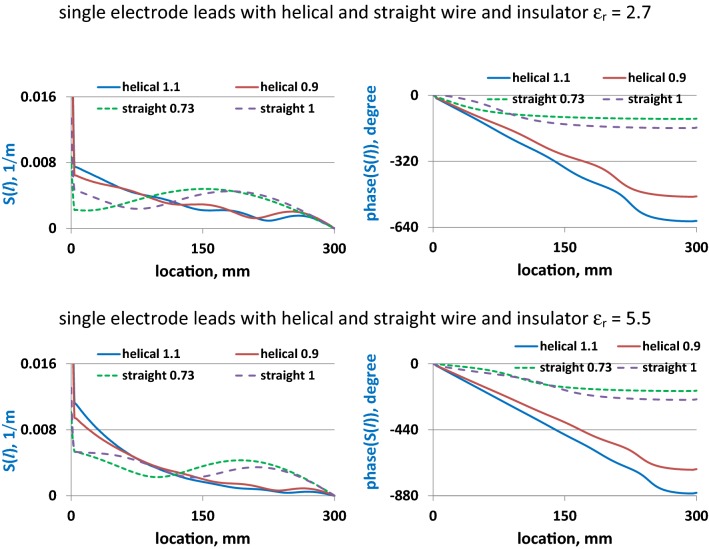
Amplitudes and phases of *S*(*l*) for single-electrode leads with the straight and helical wires in the blood medium. The leads with straight wire diameters of 0.73 and 1.0 mm are labeled as “straight 0.73” and “straight 1”, respectively. The leads with helical structures consisted of 0.1 × 0.1 mm rectangular wire external diameters of 0.9 mm and 1.1 mm are labeled as “helical 0.9” and “helical 1.1”, respectively

As shown in Figs. 4 and 5, *S*(*l*) varies substantially with changes in wire geometry and surrounding medium for all electrodes. Electrode location also significantly affected *S*(*l*) for both straight and helical leads. For the same electrode, a change of surrounding medium primarily resulted in a phase variation of *S*(*l*) along the lead length. Changing the lead insulator *ε*_r_ significantly influenced the amplitude and phase shapes of *S*(*l*) for all media. Also, increasing *ε*_r_ resulted in higher *S*(*l*) phase at all locations along both leads. Similar behavior of *S*(*l*) phases was observed for the single-electrode lead (Fig. 6).

**Fig. 7:**
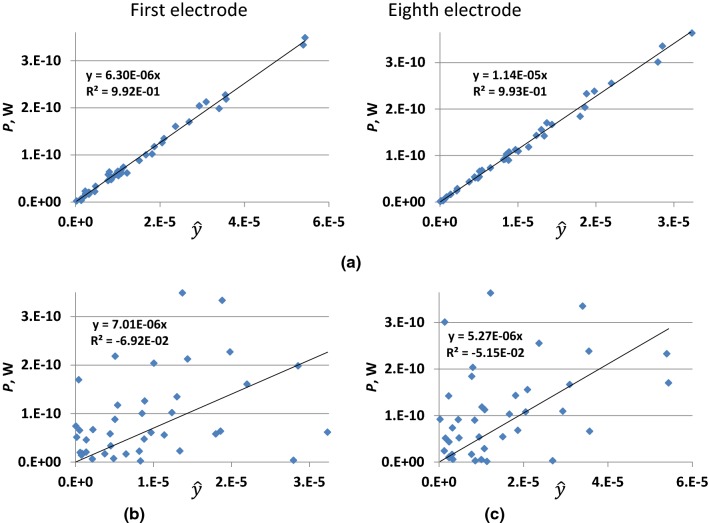
TF validation for the lead with helical wire and insulator *ε*_r_ = 2.7 surrounded by blood. **a** The TF of the corresponding electrode was used. **b** The TF of the eighth electrode was used. **c** The TF of the first electrode was used

The TF was successfully validated for all electrodes, as demonstrated by R^2^ values close to 1 (Table 2). The lowest values for R^2^ were observed for the first electrode of the lead with straight wire and insulator *ε*_*r*_ = 5.5. The applied set of *E*_tan_(*l*) ensured more than 20 dB dynamic range of *P* for all electrodes (Fig. 7a). As expected, R^2^ was close to zero if one electrode was used for TF calculation, but validation was performed for a different electrode (Fig. 7b, c). Thus, the LEM based on the TF developed for one electrode cannot be used to estimate *P* and Δ*T* for another electrode.

**Table 2:** Summary of TF validation and the scaling factor evaluation for the multi-electrode leads with straight and helical wires

Lead design	Surrounding medium	First electrode				Eighth electrode			
		R^2^	*A*, μW	*A*_T_, m·°C		*R*^2^	*A, *μW	*A*_T_, m·°C	
				10 s	200 s			10 s	200 s
Straight wire and insulator *ε*_*r*_ = 2.7	Blood	0.961	9.81	0.514	1.466	0.998	10.96	0.485	1.154
	Generic tissue	0.977	8.86	0.477	1.294	0.998	3.51	0.168	0.486
	Nerve tissue	0.989	7.42	0.470	1.337	0.999	3.04	0.166	0.529
Straight wire and insulator *ε*_*r*_ = 5.5	Blood	0.955	7.19	0.386	1.084	0.968	11.41	0.508	1.205
	Generic tissue	0.963	9.31	0.505	1.362	0.990	4.34	0.211	0.601
	Nerve tissue	0.979	7.22	0.461	1.305	0.975	5.65	0.310	0.992
Helical wire and insulator *ε*_*r*_ = 2.7	Blood	0.992	6.30	0.367	0.938	0.993	11.37	0.577	1.029
	Generic tissue	0.995	7.07	0.418	1.031	0.996	3.96	0.203	0.575
	Nerve tissue	0.996	5.94	0.390	1.076	0.999	2.94	0.163	0.519
Helical wire and insulator *ε*_*r*_ = 5.5	Blood	0.990	3.21	0.192	0.482	0.995	3.96	0.205	0.362
	Generic tissue	0.994	4.39	0.262	0.645	0.997	4.04	0.210	0.592
	Nerve tissue	0.996	4.34	0.292	0.785	0.976	2.40	0.135	0.427

The calibration factors are also presented in Table 2. The sensitivity of the calibration factor of all electrodes to the dielectric properties of the surrounding medium was significantly higher than the sensitivity of the TF for these electrodes. Dependencies of the calibration factor on the media and the electrode number were substantially different for the lead with straight and helical wires. If the surrounding media was changed from blood to nerve tissue, *A* decreased by as much as 25% and 75% for the first and eighth electrode, respectively. Variation of *A*_*T*_ was smaller than the variation of *A* for these same cases, for example for the eighth electrode the maximum decrease was 55% (versus 75% for *A*). If the surrounding media was changed from the global average to nerve tissue, *A* decreased but *A*_*T*_ increased or remained practically unaffected. This result can be attributed to differences in the thermal properties of the global average and nerve tissues. Thus, the evaluation of both the TF and the calibration factors, i.e., *A* and *A*_*T*_, should be performed for the entire lead parameter matrix.

We observed a significant dependence of *A* and *A*_*T*_ on the surrounding media, with *A* showing a variation of up to 3.9 and *A*_*T*_ of up to 2.2 for the range of electrical properties used, i.e., permittivity *ε*_r_ ranging from 44 to 78 and conductivity *σ* ranging from 0.354 to 1.2 S/m. Such a large variation in the net dissipated electrode power *P* and the net temperature rise Δ*T* makes it difficult to define the electrical properties of the surrounding media conservatively. Further investigations are necessary to propose conservative surrounding media properties.

**Fig. 8:**
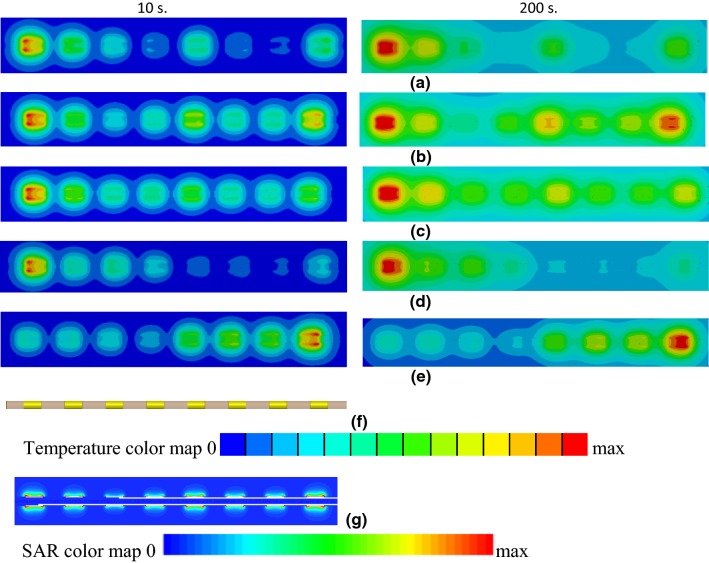
Temperature profiles in close proximity to electrodes of the lead with straight wire for RF exposure at 128 MHz after 10 s (left column) and 200 s (right column). Results are presented for leads surrounded by **a** blood, **b** generic tissue, and **c** nerve tissue. **d**, **e** Temperature profiles of 8 electrodes for RF exposure of the lead with helical wire in blood at 128 MHz for two different excitations. **f** Close-up view of the model with the electrodes. **g** SAR in close proximity to electrodes of the lead with straight wire in blood for an RF exposure of 128 MHz

Regarding the RF exposure, the temperature distributions in close proximity to the electrodes were visibly asymmetric along the electrode length, especially for exposure times less than 100 s (Fig. 8). For an exposure time of 200 s, and only for a surrounding medium of nerve tissue, the regions of highest temperature were cylindrical in nature. The temperature distributions took on the shape of a droplet for all other cases. A rapid decrease of the SAR in close proximity to the electrodes was also observed (Fig. 8g).

**Fig. 9:**
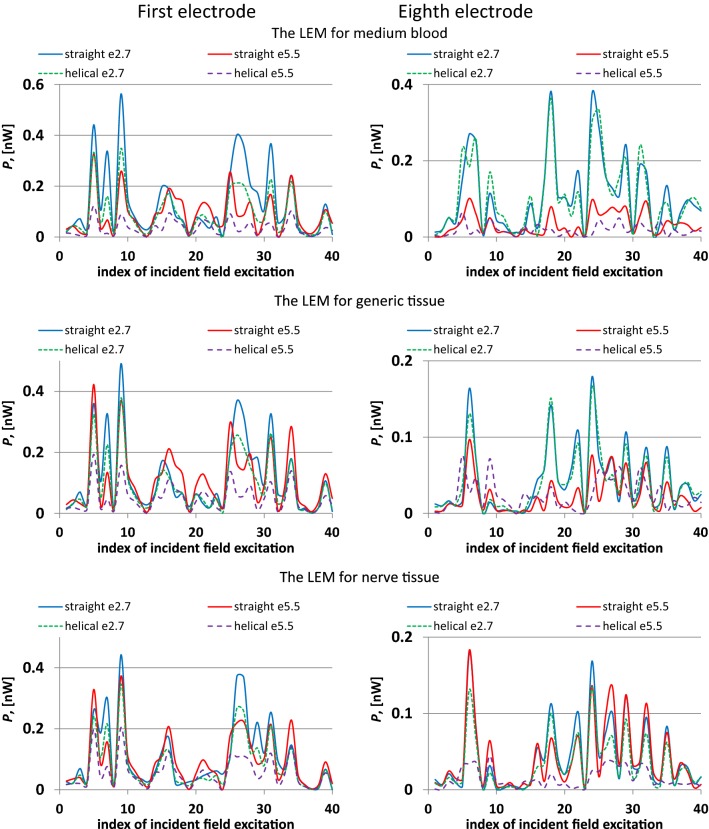
*P* versus index of incident field excitation for different lead geometries, insulator *ε*_r_, and surrounding media

The results for the influence of lead design and surrounding medium on *P* are presented in Fig. 9. Use of the artificial set of 40 *E*_tan_(*l*) showed that it is impossible to choose a lead design that always results in either the highest or the lowest combinations of *P* and Δ*T*. Therefore, it is impossible to compare *P* and Δ*T* for clinically relevant *E*_tan_(*l*) based only on known variations of |*S*(*l*)|, *A*, and *A*_*T*_ versus surrounding media. Comparing only these quantities for different surrounding media can result in the incorrect selection of the conservative tissue simulating medium, i.e., a medium that results in the largest heating for a given AIMD lead.

**Fig. 10:**
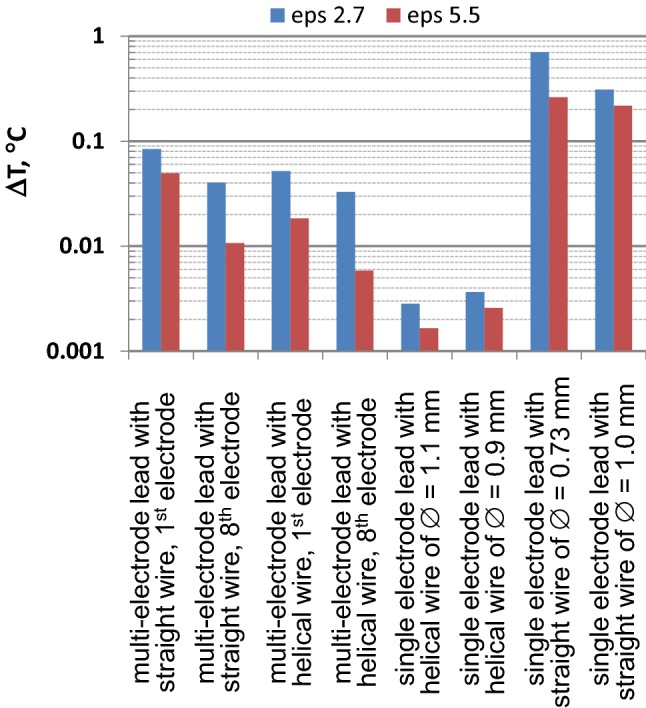
Comparison of maximum ∆*T* observed for 40 *E*_tan_(l) in blood

Comparison of the maximum Δ*T* of the 8-electrode and single-electrode leads for the 40 *E*_tan_(*l*) is presented in Fig. 10. As seen in the figure, the Δ*T* for single-electrode leads containing helical wire was significantly lower than the Δ*T* for all multi-electrode leads. Additionally, the Δ*T* for the single-electrode leads containing straight wire was significantly higher than for all multi-electrode leads. Thus, single-electrode leads cannot be used for “generic” evaluation of multi-electrode leads.

Hot spots around the leads, i.e., the points of high power deposition and temperature rise, were not determined as a separate step in our workflow. Instead, it was more straightforward and even faster to evaluate the LEM for all electrodes without first spending a substantial amount of time to determine the hot spots of each lead. Hot spot evaluation was performed concurrent with the sensitivity analysis. All electrodes were selected as hot spots in all investigated media.

### Results for power injection into leads

**Fig. 11:**
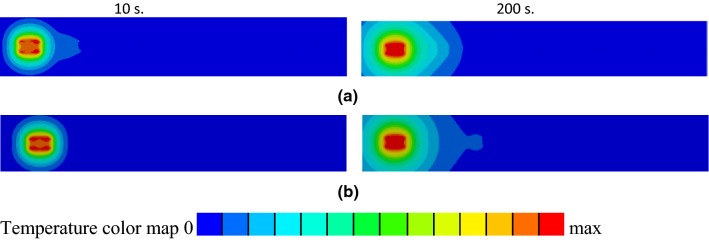
Temperature profiles for 8 electrodes for injection power into the first wire pin of the lead with straight wire and medium blood. **a** 128 MHz. **b** 0.5 MHz

For power injection into the leads with straight wire at 128 MHz and 0.5 MHz, the temperature distribution in close proximity to the electrodes was cylindrical in shape for all exposure time steps (Fig. 11). Temperature profiles for the power injection at 0.5 MHz were similar for the leads with straight and helical wires to within 3% variation. The power injection into the leads with helical wire at 128 MHz was not modeled because inductance losses were very high.

**Fig. 12:**
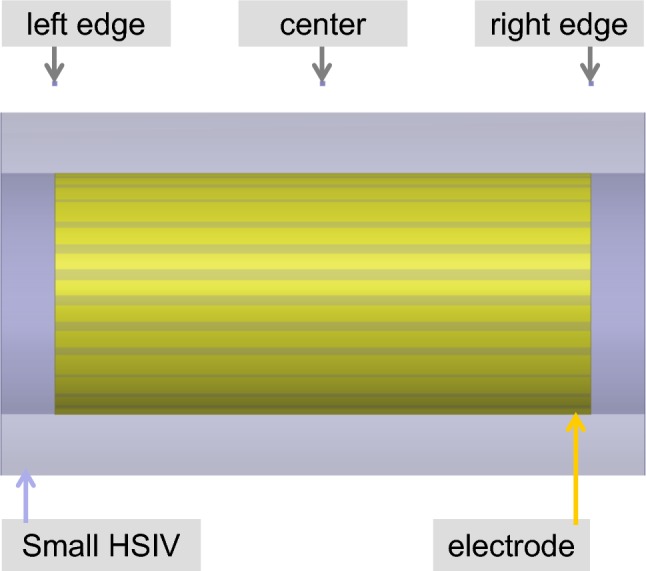
Close-up view of one electrode with locations of point temperature sensor. Center, left edge and right edge locations are 0.5 mm away from the electrode

Six quantities of interest were selected for making quantitative comparisons of the temperature distributions in close proximity to the electrodes: maximum transient temperature rise of the electrode (“electrode max”) and in a small HSIV (“small HSIV max”), minimum temperature rise in a small HSIV (“small HSIV min”), and transient temperature at three locations: 0.5 mm distance from the electrode in the *y*-direction and opposite electrode left end (“left edge 0.5 mm”), center (“center 0.5 mm”), and right end (“right edge 0.5 mm”) (Fig. 12).

The small HSIV included locations with a maximum distance to the electrode less than 0.35 mm. However, for evaluation of the temperature rise over the small HSIV, the minimum values were approximately 43% and 69% (Table 3) of the maximum values for *t* = 10 s and *t* = 200 s, respectively. Higher medium electrical conductivities resulted in increased differences of the temperature rise for the locations “left edge 0.5 mm” and “right edge 0.5 mm”. Also, the difference was larger for the leads with helical wire versus the lead with straight wire.

**Table 3:** Summary results of rescaled temperature rise for radiative exposure and injection test cases

Case	Temperature sensor locations								Min value over small HSIV		Max value over small HSIV	
	Electrode		Center		Left edge		Right edge					
	10 s	200 s	10 s	200 s	10 s	200 s	10 s	200 s	10 s	200 s	10 s	200 s
Straight wire radiative, blood	0.349	0.995	0.268	0.886	0.278	0.836	0.199	0.762	0.173	0.728	0.371	1.00
Straight wire radiative, generic tissue	0.375	0.998	0.307	0.904	0.297	0.827	0.240	0.796	0.213	0.764	0.390	1.00
Straight wire radiative, nerve tissue	0.351	0.998	0.292	0.909	0.285	0.830	0.228	0.809	0.203	0.780	0.369	1.00
Helical wire radiative, blood	0.388	0.992	0.282	0.863	0.294	0.829	0.206	0.728	0.176	0.690	0.405	1.00
Straight wire injection at 128 MHz, blood	0.417	0.999	0.366	0.906	0.319	0.802	0.322	0.804	0.295	0.764	0.427	1.00
Straight wire injection at 128 MHz, generic tissue	0.415	0.999	0.365	0.906	0.318	0.802	0.321	0.806	0.294	0.764	0.425	1.00
Straight wire injection at 128 MHz, nerve tissue	0.386	0.999	0.341	0.907	0.298	0.800	0.305	0.811	0.276	0.762	0.400	1.00
Straight wire injection at 0.5 MHz, blood	0.420	0.999	0.366	0.906	0.316	0.798	0.325	0.808	0.292	0.760	0.430	1.00
Straight wire injection at 0.5 MHz, generic tissue	0.421	0.999	0.365	0.905	0.316	0.797	0.326	0.809	0.292	0.759	0.431	1.00
Straight wire injection at 0.5 MHz, nerve tissue	0.391	0.999	0.342	0.906	0.297	0.797	0.308	0.811	0.275	0.759	0.404	1.00

For the power injection cases at 128 MHz and 0.5 MHz, the values of the temperature rise at the locations “left edge 0.5 mm” and “right edge 0.5 mm” were similar for *t* = 10 s and *t* = 200 s (Table 3). Changes in the injection power frequency or the surrounding medium had a negligible impact on the temperature rise quantities when normalized to the maximum electrode temperature rise at 200 s.

## Discussion

RF energy absorbed by a human subject undergoing MRI has the potential to increase the local tissue temperature, especially in the presence of an AIMD. To evaluate the risk of the thermal injury, *P* or Δ*T* or both are typically used together with the LEM and a set of *E*_tan_(*l*) that is clinically relevant for an AIMD. This study presented a computational modeling workflow for *P*, Δ*T*, and LEM of various multi-electrode lead designs using 3D EM and thermal co-simulation. LEM assessment included (i) TF calculation and validation and (ii) evaluation of a calibration factor. Our workflow is targeted at the rapid numerical evaluation of lead design to optimize performance in terms of RF-induced heating. The workflow also extends our understanding of multi-electrode leads with straight and helical wires. For example, it was found that (i) thermal hot spots can occur at all electrodes of a multi-electrode lead in close proximity to the electrode surface and (ii) results for generic single-electrode leads can significantly mislead the analysis of multi-electrode leads, facts that were previously neglected.

The thermal time constants for the electrodes analyzed herein ranged from 55 to 65 s. This is substantially smaller than the maximum thermal simulation time of 200 s. We also tested that the shape of the temperature distribution around the electrodes did not change for times greater than 200 s. Based on these results, it is sufficient to use a thermal exposure time that is approximately three times longer than the thermal time constant to compare different lead designs. Our workflow can accommodate shorter or longer exposure times if Δ*T* for a particular MRI sequence has to be evaluated.

The work summarized in this paper does not include measurement data. Indirect measurement is required to acquire *P* because reliable 3D volume dosimetric, i.e., specific absorption rate (SAR)*,* measurement in close proximity to an AIMD electrode is currently not feasible. This is primarily because (i) the electrode diameter is smaller than the field probe tip diameter for most AIMD leads and (ii) the spatial electric field decay is very steep and non-linear. Experimental evaluation of *P* and Δ*T* includes uncertainties related to the experimental setup (e.g., probe locations relative to the electrode, reproducibility of the incident RF exposure, etc.), as well as sensor-related uncertainties. Some types of optical temperature sensors, primarily luminescence-based sensors, can be used for Δ*T* evaluation in an RF exposure environment. The smallest tip diameter of a commercially available fiber optic temperature sensor with an unprotected sensitive element is 0.3 mm, for example the Lsens-B (Rugged Monitoring, Quebec City, QC, Canada). Such probes must be handled with care, which complicates the process of precisely positioning the probe tip in close proximity to the electrode. Another more common option are fiber optic temperature sensors with (i) a sensitive element located at the tip of the probe and with external diameter of more than 0.7 mm, for example the Lsens-P (Rugged Monitoring, Quebec City, QC, Canada) and (ii) a sensitive element coated with a plastic and having an external diameter of more than 0.5 mm, for example the Luxtron Model STB (Lumasense, Santa Clara, CA). These sensors report a spatial average value for the temperature rise with a typical relative temperature accuracy of ± 0.2 °C. Considering the limitations, current thermal measurement technologies make the collection of experimental data at the required resolution of 0.2 mm difficult to obtain and inaccurate. Therefore, verification and validation best practices were identified and followed to ensure the reliability of the simulation outputs for this application.

Regarding software quality assurance, these studies relied on commercial off-the-shelf EM (ANSYS HFSS) and thermal solvers (ANSYS NLT). Both solvers are developed, tested, and released under an ISO-certified quality management system [31]. Without access to the source code, users must in general rely on this certification process to ensure a robust quality assurance process is in place. This helps to ensure the integrity of the source code and executables from release to release. Calculation verification intends to minimize the impact of numerical solver error, discretization error, and use error on the simulation outputs [32]. Numerical solver error refers to the sensitivity of the simulation outputs to the various solver parameters associated with a given simulation, while an evaluation of discretization error ensures that the solution has been sufficiently discretized both in space and time. The spatial convergence of the EM results is ensured by the mesh adaption procedure in HFSS, while the convergence of the thermal results was ensured through manual spatial and temporal mesh convergence. Finally, use error refers to mistakes in the inputs to a computational model. All model inputs were checked visually for accuracy before performing each simulation.

Of course, using a verified modeling workflow does not guarantee that the results accurately describe the behavior of a given AIMD. This is because modeling results depend on a large set of input parameters, i.e., lead geometry, AIMD input impedance, and electrical and thermal properties of the AIMD materials. A number of assumptions and simplifications are also made when constructing the numerical model. The verified modeling workflow is useful for uncovering sensitivities of the RF responses of an AIMD to different design and environmental factors, however. And using a reverse engineering approach, an AIMD model can be adjusted to represent the behavior of a given AIMD.

In our previous study of a helical lead with a single electrode [33], a non-linear dependence of *P* and the LEM on a small volume of fat tissue surrounding the lead tip was observed. Thus, using LEMs estimated for a homogeneous medium can yield large systematic errors in estimating *P*, and consequently, the in vivo AIMD lead electrode RF-induced heating. Because it is not required by ISO/TS 10974 Tier 3 Clause #8, it was beyond the scope of this study to provide an evaluation of a multi-electrode lead LEM for a multi-tissue media. The developed numerical workflow can accommodate a multi-tissue environment, however. Future studies should investigate the LEM for other lead and electrode geometries and account for the presence of multiple tissue types surrounding the lead.

Our case study is only a first step in the complex assessment of RF-induced heating for multi-electrode leads. Future research should include, but not be limited to, the following topics: (i) comprehensive uncertainty analysis of LEM-related quantities, (ii) inclusion of temperature probes in the numerical domain, and (iii) reverse engineering of realistic multi-electrode leads.

## Conclusion

We successfully developed a numerical workflow for modeling the LEM and the RF responses of realistic multi-electrode leads containing either helical or straight wires. Our workflow is targeted to the R&D of lead design to obtain better performance in terms of RF-induced heating, and better understanding the nature of lead interactions with incident EM fields. The workflow is not expected to be used on its own for clinical trial approval or commercial device clearance. The LEM, *P* and Δ*T* were obtained through detailed modeling of the entire lead and electrodes. Our study showed that (i) all electrodes of a multi-electrode lead can be thermal hot spots, (ii) the LEMs of individual electrodes can vary substantially, (iii) the LEM should be evaluated for each electrode, and (iv) the usage of results for generic single-electrode leads can significantly mislead the analysis of multi-electrode leads.

The outcome of this study cannot be readily generalized to the conclusion that the TF for any multi-electrode lead, independent of lead and electrode geometry, can be validated with R^2^ > 0.95. Credible numerical or experimental LEM estimation should be carried out for each lead and electrode geometry to ascertain the LEM uncertainty. In other words, findings obtained from this numerical workflow may need to be confirmed by measurements before getting clinical trial approval or commercial device clearance.
